# Modulation of salt tolerance in Thai jasmine rice (*Oryza sativa* L. cv. KDML105) by *Streptomyces venezuelae* ATCC 10712 expressing ACC deaminase

**DOI:** 10.1038/s41598-018-37987-5

**Published:** 2019-02-04

**Authors:** Suranan Yoolong, Worarat Kruasuwan, Huyền Thị Thanh Phạm, Ratchaniwan Jaemsaeng, Chatchawan Jantasuriyarat, Arinthip Thamchaipenet

**Affiliations:** 1 Department of Genetics, Faculty of Science, Kasetsart University, Bangkok, Thailand; 2 Present Address: Mitrphol Innovation and Research Center, Chaiyaphum, Thailand

## Abstract

1-Aminocyclopropane-1-carboxylate (ACC) deaminase is a plant growth promoting (PGP) trait found in beneficial bacteria including streptomycetes and responsible for stress modulation. The ACC deaminase gene, *acdS*, of *S*. *venezuelae* ATCC 10712 was cloned into an expression plasmid, pIJ86, to generate *S*. *venezuelae*/pIJ86-*acdS*. Expression of *acdS* and production of ACC deaminase of *S*. *venezuelae*/pIJ86-*acdS* were significantly higher than the unmodified strain. The ACC deaminase-overexpressing mutant and the wild type control were inoculated into Thai jasmine rice (*Oryza sativa* L. cv. KDML105) under salt stress conditions. *S*. *venezuelae* on its own augmented rice growth and significantly increased more tolerance to salinity by reduction of ethylene, reactive oxygen species (ROS) and Na^+^ contents, while accumulating more proline, total chlorophyll, relative water content (RWC), malondialdehyde (MDA), and K^+^ than those of uninoculated controls. The overproducer did not alter chlorophyll, RWC, or MDA further–while it did boost more shoot weight and elongation, and significantly regulated salt tolerance of rice by increasing proline and reducing ethylene and Na^+^ contents further than that of the wild type. This work is the first illustration of the beneficial roles of *S*. *venezuelae* to enhance plant fitness endophytically by promotion of growth and salt tolerance of rice.

## Introduction

Soil salinity in arid regions is often an important limiting factor for cultivation of agricultural crops such as maize, rice, and sugarcane. Excess of salt affects plant growth by increasing stress factors, such as ethylene production, Na^+^ accumulation, and reactive oxygen species (ROS) which is detrimental to the plant’s physiology, leading to growth impairment^1–3^.

Streptomycetes have been recognized recently as plant growth promoting (PGP) bacteria that can protect plants from infectious diseases and enhance plant growth through several PGP-traits, such as siderophore production, plant hormone production, and phosphate solubilization^4–6^. Furthermore, PGP-bacteria assist plants to grow under severe condition caused by drought, flooding, salinity, and phytopathogens by the action of 1-aminocyclopropane-1-carboxylate (ACC) deaminase^7–11^. ACC deaminase, encoded by the *acdS* gene, is responsible for the breakdown of ACC, which is the direct precursor of ethylene in all higher plants, into ammonia and α-ketobutyrate - which bacteria consume as nitrogen and carbon sources^12^. Overexpression of *acdS* in endophytic bacteria remarkably improved plant growth and alleviated stresses in plants, when compared to uninoculated plants and those of wild type inoculation. For example, ACC deaminase-overproducing strains of *Pseudomonas putida* ameliorated flooding stress in tomato^13^, *Sinorhizobium meliloti* improved growth and copper tolerance in *Medicago lupulina*^14^, and *Serratia grimesii* enhanced growth and the level of plant protection against seed-borne pathogens in the common bean^15^.

*Streptomyces venezuelae* was discovered from soil and, thus far, has been known as a cell factory for the production of diverse natural products including chloramphenicol, watasemycin, and venemycin^16–18^. Although the genome sequence of *S*. *venezuelae* was determined and characterized^19^, the information was used mainly for investigation of gene clusters involved in antibiotic biosynthesis. The genome sequence has never been inspected for a role of plant-beneficial functions; likewise, *S*. *venezuelae* has never been documented as a PGP-endophytic bacterium. Recently, genes contributing to PGP-traits including *acdS* were not only present in genome sequences of PGP-rhizobacteria (PGPR) but also found in those of non-PGPR^20,21^.

On this basis, we examined genes related to PGP-function in all genome sequences of members of a genus *Streptomyces* available in the GenBank database, in particular *acdS*. Surprisingly, *acdS* was present in many genomes of non-PGP-endophytic *Streptomyces*, including *S*. *venezuelae*. To address the possible beneficial role of *S*. *venezuelae* interacting beneficially with plants and modulating salt stress, *S*. *venezuelae* was inoculated into the salt-sensitive Thai jasmine rice KDML105 cultivar. Furthermore, the effects of overexpression of *acdS* within *S*. *venezuelae* towards rice growth and salt tolerance were investigated. The physiology of rice associated with *S*. *venezuelae* and its overexpressed mutant under salt stress condition are discussed.

## Results

### Salt tolerance and PGP-traits of *S*. *venezuelae*

Analysis of salt tolerance of *S*. *venezuelae* ATCC 10712 revealed that it had tolerated NaCl up to 3% (w/v). During growth in 3% NaCl, proline was accumulated significantly, at 36.66 ± 0.24 µM in cells (Supplementary Table S1). Moreover, *S*. *venezuelae* had ACC deaminase activity of 364.21 ± 19.28 nmol α-ketobutyrate mg protein^−1^ h^−1^ and produced IAA at 21 ± 1.02 μg mL^−1^ (Supplementary Table S1).

### Characterization of ACC deaminase-overexpressing *S*. *venezuelae*

**Figure 1:**
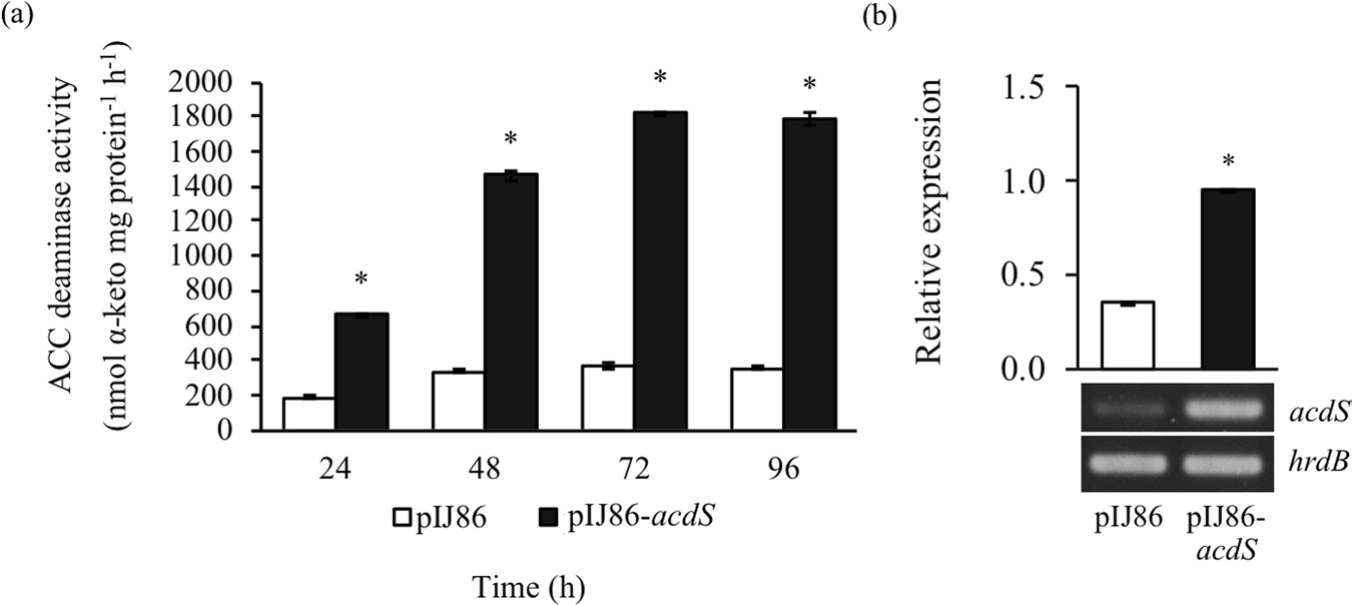
ACC deaminase activity (**a**) and semi-quantitative RT-PCR analysis of expression of *acdS* (**b**) of *S*. *venezuelae*/pIJ86 (pIJ86) and *S*. *venezuelae*/pIJ86-*acdS* (pIJ86-*acdS*). The values represent the mean ± S.E. of three replicates and an asterisk (*) indicate statistically significant changes in expression (t test, *p* < 0.05).

An ACC deaminase-overexpressing mutant, *S*. *venezuelae*/pIJ86-*acdS*, was constructed and verified by resistance to apramycin and thiostrepton. The wild type with empty plasmid, *S*. *venezuelae*/pIJ86, was also constructed as a control. In comparison with *S*. *venezuelae*/pIJ86, ACC deaminase activity of *S*. *venezuelae*/pIJ86-*acdS* was enhanced 5-fold from 72–96 h of incubation in MM containing 3 mM ACC (Fig. 1a, Supplementary Table S2). This result correlated with the high expression profile of *acdS* by *S*. *venezuelae*/pIJ86-*acdS* at 72 h (2.7-fold) when compared to that of wild type control (Figs 1b and S1 and Table S3). The ACC deaminase activity of *S*. *venezuelae*/pIJ86-*acdS* were relatively stable when re-streaked for up to 5 generations without antibiotic selection (data not shown).

### Plant colonization and growth promotion by *S*. *venezuelae*

**Figure 2:**
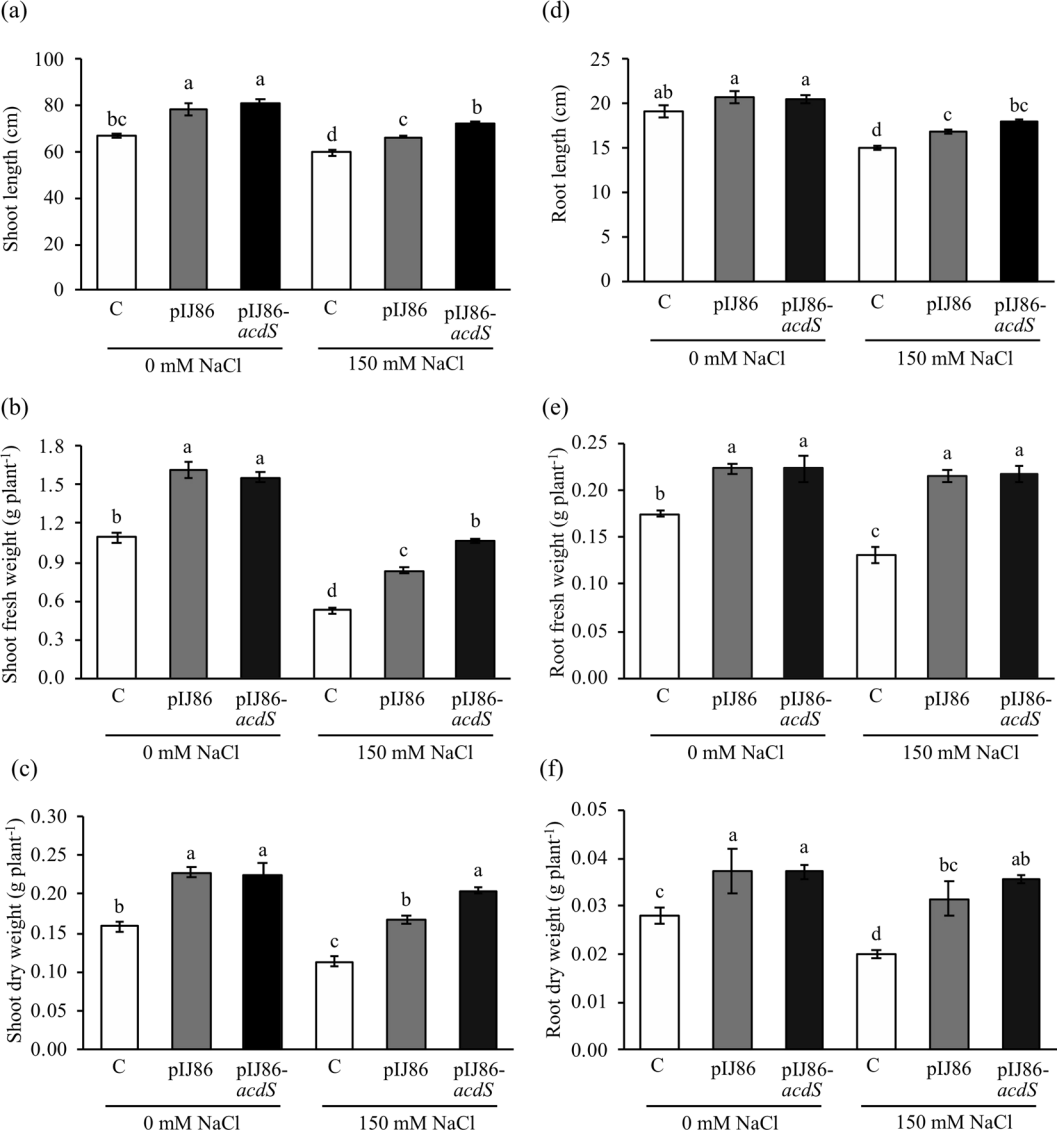
Effect of ACC deaminase-producing *Streptomyces venezuelae* on shoot length (**a**), shoot fresh weight (**b**), shoot dry weight (**c**), root length (**d**), root fresh weight **(e**), and root dry weight (**f**) of rice plants under non-salt (0 mM NaCl) and salt stress (150 mM NaCl) conditions. The values show the mean ± S.E. of twelve replicates and bars carrying different letters are significantly different (Tukey’s test, *p* < 0.05). C, uninoculated rice control; pIJ86, rice inoculated with *S*. *venezuelae/*pIJ86; pIJ86-*acdS*, rice inoculated with *S*. *venezuelae*/pIJ86-*acdS*.

*S*. *venezuelae*/pIJ86 was successfully inoculated into Thai jasmine rice cv. KDML105 grown under hydroponic condition with and without salt treatment. *S*. *venezuelae*/pIJ86 was re-isolated from rice under both treatments at about 10^4^ CFU g root fresh weight^−1^ (Supplementary Table S4) indicating that *S*. *venezuelae* had the ability to colonize inside plants. In addition, un-inoculated plants did not harbor any streptomycete (data not shown) showing that rice seeds were surface sterilized effectively, and the hydroponic conditions used in this study were free from contamination. Growth parameters of inoculated and uninoculated rice KDML105 were evaluated at 7 days after being treated with and without 150 mM NaCl. In comparison to uninoculated plants, rice associated with *S*. *venezuelae*/pIJ86 had significant increases of shoot/root lengths and shoot/root fresh/dry weights in both non-salt and salt treatments (Fig. 2a–f).

### Effect of overexpression of ACC deaminase on plant growth parameters

Growth parameters including shoot/root length and shoot/root fresh/dry weights of rice KDML105 inoculated with *S*. *venezuelae*/pIJ86 were enhanced significantly, when compared with uninoculated plants in both non-salt and salt treatments (Figs 2a–f and S2). Similar to the original strain, its overexpressing mutant, *S*. *venezuelae*/pIJ86-*acdS*, greatly promoted growth of rice in both non-salt and salt stress conditions (Figs 2a–f and S2), but highly increased shoot length and biomass in particular more than those inoculated with wild type control under salt stress conditions (Fig. 2a–c).

### Effect of overexpression of ACC deaminase on plant ethylene

**Figure 3:**
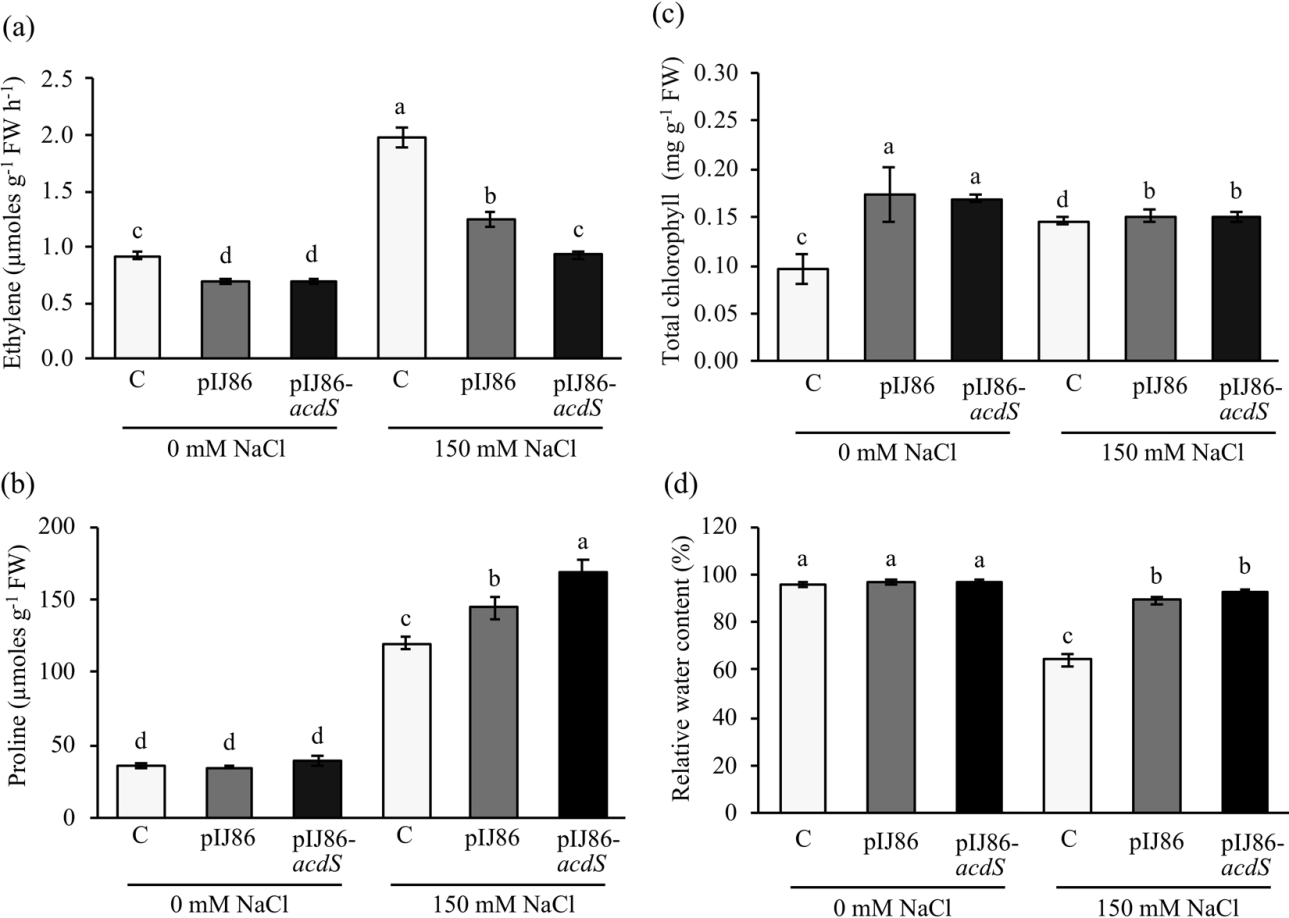
Effect of ACC deaminase-producing *Streptomyces venezuelae* on ethylene (**a**), proline (**b**), total chlorophyll (**c**), and relative water contents (RWC) (**d**) of rice plants under non-salt (0 mM NaCl) and salt stress (150 mM NaCl) conditions. The values show the mean ± S.E. of twelve replicates and bars carrying different letters are significantly different (Tukey’s test, *p* < 0.05). C, uninoculated rice control; pIJ86, rice inoculated with *S*. *venezuelae/*pIJ86; pIJ86-*acdS*, rice inoculated with *S*. *venezuelae*/pIJ86-*acdS*.

At 7 days after irrigation with 150 mM NaCl, the ethylene level of uninoculated plants was increased about 2-fold when compared with those grown without salt (Fig. 3a, Supplementary Table S4). When rice was associated with *S*. *venezuelae*/pIJ86, the ethylene level was reduced 1.6-fold when compared to uninoculated plants (Fig. 3a, Supplementary Table S4). When rice was inoculated with *S*. *venezuelae*/pIJ86-*acdS*, the ethylene level was decreased 2-fold when compared to uninoculated plants (Fig. 3a, Supplementary Table S4). The results indicated that overexpression of ACC deaminase facilitated salt tolerance in plants by reduction of ethylene to the same level as that of the non-salt treatment control.

### Effect of overexpression of ACC deaminase on proline content

Under non-salt conditions, proline content was unaffected in rice KDML105 inoculated with either *S*. *venezuelae*/pIJ86 or *S*. *venezuelae*/pIJ86-*acdS* (Fig. 3b). Under salt-stress conditions, the proline content was 3.4-fold higher in uninoculated plants compared to those grown in non-salt conditions (Fig. 3b, Supplementary Table S4). Nonetheless, the proline content of plants associated with *S*. *venezuelae*/ pIJ86 was increased significantly compared to the uninoculated control and even higher in plants inoculated with *S*. *venezuelae*/pIJ86-*acdS* (Fig. 3b). The results demonstrated that overexpression of ACC deaminase facilitated salt tolerance in rice by escalation of proline content.

### Effect of overexpression of ACC deaminase on total chlorophyll and RWC

The total chlorophyll content of rice KDML105 inoculated with either *S*. *venezuelae*/pIJ86 or *S*. *venezuelae*/pIJ86-*acdS* (1.8-fold) was augmented significantly when compared to uninoculated plants under non-salt conditions (Fig. 3c, Supplementary Table S4). Under salt stress conditions, rice associated with either the wild-type control or the ACC deaminase-overexpressing mutant maintained a higher chlorophyll content compared to that of uninoculated rice (Fig. 3c). RWC of uninoculated plants under salt treatment was 1.5-fold decreased when compared to untreated controls (Fig. 3d, Supplementary Table S4). Significantly, RWC in rice associated with either *S*. *venezuelae*/pIJ86 or *S*. *venezuelae*/pIJ86-*acdS* was 1.4-fold higher when compared to the uninoculated control. The results suggested that *S*. *venezuelae* induced salt tolerance in rice by elevation of chlorophyll content and RWC.

### Effect of overexpression of ACC deaminase on Na^+^ and K^+^ contents

**Figure 4:**
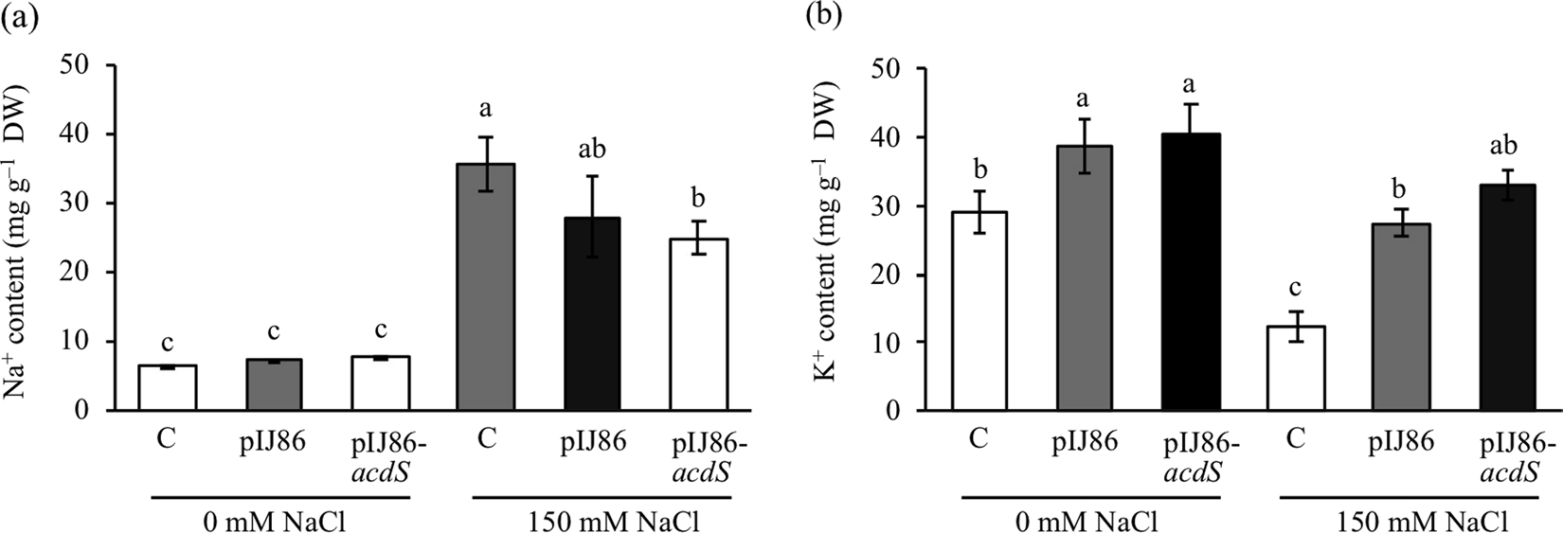
Effect of ACC deaminase-producing *Streptomyces venezuelae* on Na^+^ (**a**) and K^+^ (**b**) contents of rice plants under non-salt (0 mM NaCl) and salt stress (150 mM NaCl) conditions. The values show the mean ± S.E. of twelve replicates and bars carrying different letters are significantly different (Tukey’s test, *p* < 0.05). C, uninoculated rice control; pIJ86, rice inoculated with *S*. *venezuelae/*pIJ86; pIJ86-*acdS*, rice inoculated with *S*. *venezuelae*/pIJ86-*acdS*.

When rice was grown under salt stress conditions, Na^+^ was accumulated up to 56-fold compared to that of non-salt treatment (Fig. 4a, Supplementary Table S4). Significantly, the Na^+^ content in rice inoculated with either *S*. *venezuelae*/pIJ86 or *S*. *venezuelae*/pIJ86-*acdS* decreased about 1.4-fold when compared to uninoculated rice (Fig. 4a, Supplementary Table S4). On the contrary, the K^+^ content decreased (2.3-fold) when rice was grown under salt stress conditions (Fig. 4b). However, rice inoculated with either *S*. *venezuelae*/pIJ86 or *S*. *venezuelae*/pIJ86-*acdS* had significantly increased K^+^ content under both non-salt and salt treatments (Fig. 4b, Supplementary Table S4). Markedly, rice inoculated with the ACC deaminase-overexpressing mutant had the highest significant increase in K^+^ content by 2.6-fold when compared to the uninoculated control under salt stress conditions (Fig. 4b, Supplementary Table S4). The results demonstrated that overexpression of ACC deaminase helped salt tolerance in rice by reduction of Na^+^ content, and increase in K^+^ content.

### Effect of overexpression of ACC deaminase on ROS

**Figure 5:**
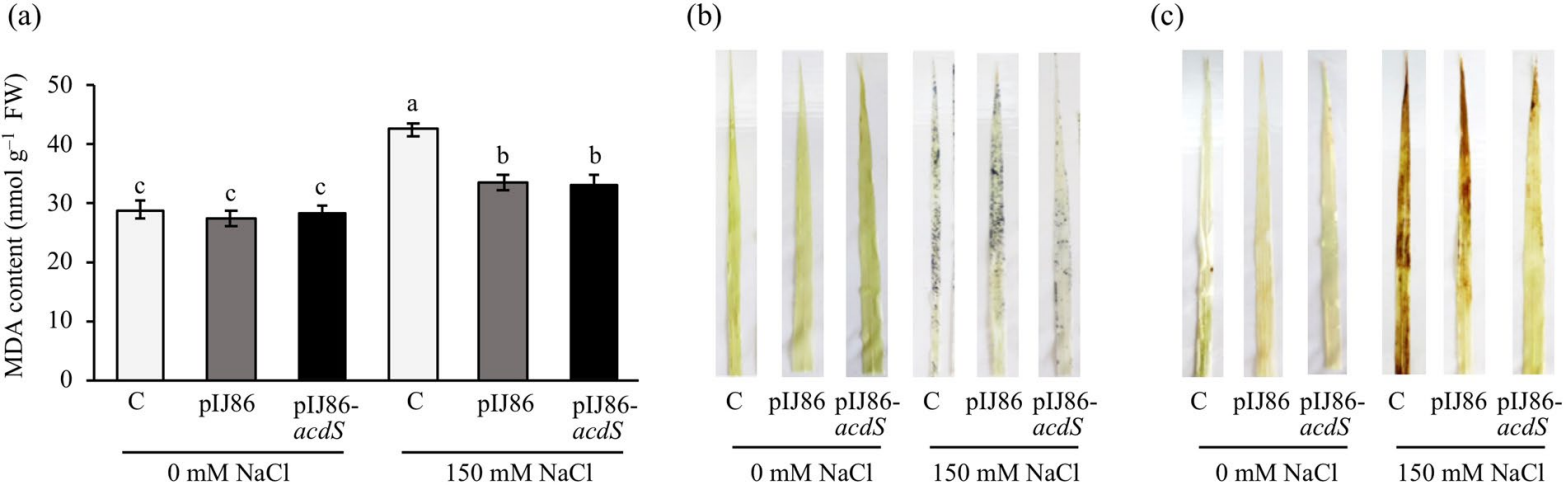
Effect of ACC deaminase-producing *Streptomyces venezuelae* on MDA content (**a**), histochemical NBT staining (**b**), and DAB staining (**c**) of rice plants under non-salt (0 mM NaCl) and salt stress (150 mM NaCl) conditions. The values indicate the mean ± S.E. of twelve replicates and bars carrying different letters are significantly different (Tukey’s test, *p* < 0.05). C, uninoculated rice control; pIJ86, rice inoculated with *S*. *venezuelae/*pIJ86; pIJ86-*acdS*, rice inoculated with *S*. *venezuelae*/pIJ86-*acdS*.

Salt stress drastically induced lipid peroxidation. The MDA content was increased up to 1.5-fold in rice grown under salt stress conditions (Fig. 5a, Supplementary Table S4). However, rice KDML105 inoculated with either *S*. *venezuelae*/pIJ86 or *S*. *venezuelae*/pIJ86-*acdS* had a significant reduction in MDA content - about 1.3-fold when compared to the uninoculated control (Fig. 5a, Supplementary Table S4). ROS in leaves were detected by the presence of superoxide and hydrogen peroxide by staining with nitrobluetrazolium (NBT) (Fig. 5b) and 3,3′-diaminobenzidine (DAB) (Fig. 5c), respectively. In the presence of salt, both ROS species were present, shown by the intense staining of leaves; however rice inoculated with *S*. *venezuelae*/pIJ86 or *S*. *venezuelae*/pIJ86-*acdS* showed fainter staining than those of the uninoculated control (Fig. 5b,c). The results indicated that *S*. *venezuelae* helped salt tolerance in rice by reduction of MDA content and ROS species. However, under salt stress conditions, the overexpression of ACC deaminase did not induce those characteristics more than those of the wild type control.

## Discussion

ACC deaminase is a bacterial enzyme found in several PGP-bacteria including *Bacillus*, *Enterobacter*, *Pseudomonas* and *Streptomyces*. Its improved stress tolerance of plants to drought, flooding, salinity and phytopathogens^7–11^. Interestingly, increasing the ACC deaminase activity by overexpression of the corresponding gene in PGP-bacteria remarkably facilitated growth and alleviated environmental stresses of host plants more than those of wild type strains^13–15^.

In this work *acdS*, encoding ACC deaminase located in the genome of *S*. *venezuelae* ATCC 10712, was cloned and expressed in this strain. The overexpressing mutant, *S*. *venezuelae*/pIJ86-*acdS*, had higher ACC deaminase activity, compared to *S*. *venezuelae*/pIJ86. The results were in agreement with previous reports that overexpression of ACC deaminase in *Mesorhizobium cicero*, *Serratia grimesii* and *Sinorhizobium meliloti* resulted in higher ACC deaminase activity, compared to the corresponding wild type strains^14,15,22^. In addition, *acdS* expression under the *ermE* promoter of multi-copy plasmid pIJ86 in *S*. *venezuelae* without antibiotic selection was maintained up to 5 generations, consistent with the previous report^23^. Interestingly, *S*. *venezuelae* showed endophytic ability in rice plants; which was proven by re-isolation of the bacterium responsible for promotion of rice growth from plant tissues; a trait that is herein shown for the first time for this bacterium. Soil actinomycetes, therefore, potentially act as endophytes, supporting the hypothesis that bacterial communities in the rhizosphere, rhizoplane, and endosphere of rice root microbiomes were overlapping^24^. In this work, it was demonstrated for the first time that *S*. *venezuelae* behaves as a PGP-endophytic bacterium.

Under normal conditions, rice inoculated with *S*. *venezuelae*/pIJ86 significantly increased biomass of shoot and root, and elongation. This might be due to an action of IAA produced by this strain that would encourage plant growth and elongation. Moreover, the results were in agreement with previous work showing that ACC deaminase-producing *Streptomyces* have the ability to enhance growth of *Jatropha curcas*, mung bean, sugarcane, and rice^6,8,10,25^. Apart from *Streptomyces*, IAA and ACC deaminase-producing species from the genera *Agromyces*, *Bacillus*, *Enterobacter*, *Methylophaga*, *Microbacterium*, *Paenibacillus*, and *Pseudomonas* were also reported to promote growth of canola, rice, sugarcane, and tomato^26,27^.

Under salt stress conditions, rice plants inoculated with either *S*. *venezuelae*/pIJ86 or its overexpressing mutant, *S*. *venezuelae*/pIJ86-*acdS*, showed enhanced growth parameters compared to those of uninoculated controls. However, shoot length and biomass of rice associated with the ACC deaminase-overexpressing mutant were significantly greater than plants inoculated with the unmodified strain. Our results were in congruence with other studies in which ACC deaminase overexpressing strains of *Pseudomonas putida* and *Serratia grimesii* promoted growth of tomato and common bean, respectively compared to wild type strains^13,15^. Therefore, the results unambiguously demonstrated that ACC deaminase-overexpressing *S*. *venezuelae* facilitated rice growth better than the original strain under salt stress conditions.

It is generally known that ethylene production is a main response in plants exposed to environmental stress. Salinity induced a high level of ethylene via the actions of ACC synthase and ACC oxidase towards ACC, an ethylene precursor. Whereas, ACC deaminase of bacteria assists plants in responding by conversion of ACC into ammonia and α-ketobutyrate and, thus, reducing ethylene as a consequence^28^. In this work, the ethylene levels were significantly lower when rice was associated with either *S*. *venezuelae*/pIJ86 or *S*. *venezuelae*/pIJ86-*acdS* compared to that of the uninoculated control. Our results were similar to previous reports that ethylene levels in rice and sugarcane were reduced by ACC deaminase-producing *Streptomyces* sp. GMKU 336^10^ and *Enterobacter* sp. EN-21^9^ respectively, under salt stress conditions. The ethylene level was lowest in rice inoculated with the ACC deaminase-overexpressing mutant, correlating with the high ACC deaminase activity of this strain. The results were consistent with another report that overexpression of ACC deaminase in endophytic *Pseudomonas* spp. enhanced salt tolerance in tomato by reducing ethylene production^29^. In addition, a lower amount of ACC was observed in tomatoes inoculated with ACC deaminase-overexpressing psychrotolerant bacteria under chilling stress^30^.

Proline accumulation is one of the adaptation mechanisms of plants under salt stress. At 7 days after irrigation with salt, the proline content of rice associated with *S*. *venezuelae* was high and particularly higher in rice inoculated with the ACC deaminase-overexpressing mutant. The results agreed with data on ACC-deaminase producing *Dietzia natronolimnaea* and *Streptomyces* sp. GMKU 336, associated with wheat and rice respectively – which induced elevated proline content^31,32^. Accumulation of higher levels of proline stabilized proteins, cell structures, and osmotic balance^33^ in rice associated with *S*. *venezuelae*/pIJ86-*acdS* and, thus, accelerated salt tolerance.

Reduction of total chlorophyll and RWC of plants are generally the first notable effects of salt stress such as those reported in black gram and rice^10,34,35^. In this work, the total chlorophyll and RWC of rice plants were increased significantly in plants under salt treatment, when inoculated with either *S*. *venezuelae*/pIJ86 or *S*. *venezuelae*/pIJ86-*acdS*. The results were in congruence with other studies in which ACC deaminase-producing *Enterobacter* sp. SBP-6 in wheat^32^, *Enterobacter* cloacae HSNJ4 in canola^36^, *Bacillus subtilis* RJ46, *Ochrobactrum pseudogrignonense* RJ12, and *Pseudomonas* sp. RJ15 in black gram and pea^37^, and bacterial consortia in avocado^38^ increased chlorophyll level more than those of non-inoculated plants, when under salt stress. Moreover, the results were in agreement with other for the ACC deaminase-overexpressing endophytic *Pseudomonas* spp., which improved photosynthetic performance and water content in tomato^29^. The results suggested that *S*. *venezuelae* facilitates rice growth in saline environments by increasing total chlorophyll and RWC. However, as the ACC deaminase-overproducing *S*. *venezuelae* enhanced chlorophyll content and RWC equally to those of the wild type control, it can be concluded that the overexpression of ACC deaminase did not influence those characters.

Excess accumulation of Na^+^ and inhibition of K^+^ uptake under salt stress are very harmful for plant cells, leading to growth impairment^3^. Several reports have indicated that increasing the K^+^/Na^+^ ratio is crucial for salt tolerance in plants^39–41^. In this work, the Na^+^ content was significantly enhanced, while the K^+^ content was decreased drastically in salt-stressed uninoculated rice. On the contrary, rice inoculated with either *S*. *venezuelae*/pIJ86 or *S*. *venezuelae*/pIJ86-*acdS* had markedly reduced Na^+^ content and enhanced K^+^ content. The results were similar to recent reports that ACC deaminase-producing *Dietzia natronolimnaea* and *Streptomyces* sp. GMKU 336 enhanced salt tolerance in plants by increasing the K^+^/Na^+^ ratio via up-regulation of the Na^+^/H^+^ antiporter gene (*NHX1*) involved in maintenance of the Na^+^ level in the cytoplasm^10,42^. Besides, the increment in K^+^/Na^+^ ratio was observed in maize, pea, and sugarcane associated respectively with ACC deaminase-producing *Pseudomonas fluorescens*, *Variovorax paradoxus* 5C-2, and *Enterobacter* sp. EN-21, under salinity stress^9,43,44^.

ROS production plays a crucial role as signalling molecules involved in stress conditions including attack by pathogens, drought, and salinity which leads to high accumulation of MDA, a product of membrane lipid peroxidation^1,2^. In this study, rice KDML105 inoculated with either *S*. *venezuelae*/pIJ86 or *S*. *venezuelae*/pIJ86-*acdS* had significantly decreased MDA content under salt stress conditions. Moreover, histochemical staining with NBT and DAB indicated that levels of H_2_O_2_ and O^2−^ were reduced in the corresponding leaves. The results were in agreement with previous reports that ACC deaminase-producing endophytes caused a reduction in MDA content, including *Bacillus subtilis* GB03 in white clover^31^, *Enterobacter* sp. EN-21 in sugarcane^9^, *Streptomyces* sp. GMKU 336 in Thai jasmine rice^10^, and *Dietzia natronolimnaea* in wheat^42^. Moreover, higher accumulation of proline in rice associated with *S*. *venezuelae* might help stabilize ROS^33^ and, thus, alleviate salt stress by modulation of the antioxidant system.

This work is the first demonstration that *S*. *venezuelae* carries PGP-traits and promotes growth of rice KDML105 endophytically under normal and salinity conditions. Moreover, the ACC deaminase-overexpressing mutant, *S*. *venezuelae*/pIJ86-*acdS*, enhanced rice growth and salt tolerance more than the original strain. The physiology of the rice benefitted remarkably from the ACC-deaminase trait. Overproduction of ACC deaminase of *S*. *venezuelae* is an important model to investigate how excessive ACC deaminase-producing inocula can be effective for crop health improvement under severe conditions.

## Methods

### Bacterial salt tolerance and plant growth promoting (PGP) traits

*Streptomyces venezuelae* ATCC 10712 was grown and maintained on mannitol soybean agar (MS)^45^. Salt tolerance was determined by growth of colonies on ISP 2 (Difco™) supplemented with 1–4% NaCl (w/v) at 28 °C for 7 days.

Proline accumulation was determined by growing *S*. *venezuelae* in 10 mL tryptic soy broth (TSB) supplemented with 1–3% NaCl at 28 °C for 3 days. Cells were treated with 2 mL 20% trichloroacetic acid, mixed and centrifuged. The aqueous solution was mixed with 2 mL ninhydrin solution (1.25 g ninhydrin in 30 mL glacial acetic acid and 20 mL 6 M phosphoric acid) and 2 mL glacial acetic acid, and incubated at 95 °C for 1 h, then cooled on ice. The reaction mixture was extracted and mixed vigorously with 4 mL toluene for 15–20 sec. The absorbance of the red-colored organic layer of the ninhydrin-proline complex was measured at 520 nm by spectrophotometry. Proline concentration was determined from a standard curve of commercial proline and calculated as described by Bates, *et al*.^46^.

Indole-3-acetic acid (IAA) was determined by a colorimetric method^47^. *S*. *venezuelae* was grown in the dark in glucose-beef extract broth supplemented with 10 mM L-tryptophan at 28 °C for 7 days. The culture was then centrifuged and 2 mL of supernatant was mixed with 1 mL of Salkowski’s reagent^48^. The mixture was left at room temperature for 30 min in the dark. IAA production was indicated by development of a pink-red color.

ACC deaminase activity was monitored by the amount of α-ketobutyrate generated from ACC cleavage as described by Penrose and Glick^49^. *S*. *venezuelae* was cultured in TSB and washed twice before transferring onto minimal medium (MM) containing 3 mM ACC as a sole source of nitrogen and incubated on a rotary shaker in the dark for 0, 24, 48, 72 and 98 h. The amount of α-ketobutyrate was determined by measuring absorbance at 540 nm and comparing to a standard curve of α-ketobutyrate. Protein content was performed according to Bradford^50^. ACC deaminase activity was expressed as α-ketobutyrate production in nmol mg^−1^ protein h^−1^.

### Construction of ACC deaminase-overexpressing mutant

The ACC deaminase gene (*acdS*) (SVEN_RS07535) was retrieved from the genome sequence of *S*. *venezuelae* ATCC 10712 (Accession no. NC_018750). Specific primers for amplification of *acdS* were designed as ATT151F (5′-TTTTTTAAGCTTGAGATGACGGCGATGGGCGAGTT-3′) and ATT151R (5′-TTTTTTCATATGCCGACCAGCAGCCGTCACTCAAC-3′) including respectively *Hind*III and *Nde*I sites (underlined). PCR conditions were initially 98 °C, 30 sec; and 30 cycles of 98 °C, 10 sec; 69 °C, 30 sec; 72 °C, 1 min; and finally at 72 °C, 10 min. The PCR product was then cloned into the pJET cloning vector (Fermentas, USA) and subcloned into constitutive multi-copy expression plasmid pIJ86 under ermE* promoter^51^ to obtain pIJ86-*acdS*. Next, pIJ86-*acdS* was transformed into *E*. *coli* ET12567/pUZ8002^52^ and intergeneric conjugation was performed using 24-h mycelium of *S*. *venezuelae* as described by Vitayakritsirikul, *et al*.^23^. Exconjugants (*S*. *venezuelae*/pIJ86-*acdS*) were selected by apramycin (100 μg mL^−1^) and thiostrepton (50 μg mL^−1^) resistance, and verified by (i) PCR amplification of the thiostrepton resistance gene using primers and conditions as described previously by Rungin, *et al*.^5^ and (ii) ACC deaminase activity. *S*. *venezuelae*/pIJ86 was also constructed as a control.

### RNA purification and semi-quantitative RT-PCR

*S*. *venezuelae*/pIJ86 and *S*. *venezuelae*/pIJ86-*acdS* were grown in TSB for 24 h, then harvested by centrifugation, washed twice with 0.1 M Tris-HCl (pH 8.5) and inoculated onto MM medium containing 3 mM ACC and incubated for 72 h. Total RNA was isolated using TRIzol (Ambion, USA) and treated with RNase-free DNase I according to the manufacturer’s protocol (Thermo Fisher Scientific, USA). cDNA was synthesized using a RevertAid™ First Strand cDNA Synthesis Kit (Thermo Fisher Scientific, USA). Semi-quantitative RT-PCR analysis of *acdS* gene was performed using cDNA and primers, ATT165F (5′-CGGGTGATCTGCTCGTGGGTCGGTA-3′) and ATT165R (5′-GCGGGCTTCGGCATCGGCTT-3′), using Phusion Hot Start II-High Fidelity DNA polymerase (Thermo Fisher Scientific, USA). PCR conditions started with 98 °C, 30 sec; and 30 cycles of 98 °C, 10 sec; 58 °C, 30 sec; 72 °C, 1 min; and finally at 72 °C, 10 min. The expression level of *acdS* was quantified by Gel Doc^TM^ XR + with Image Lab^TM^ Software (Biorad, USA) and normalized against the expression of a housekeeping gene, *hrdB*^53^.

### Analysis of rice growth parameters

Thai jasmine rice seeds (*Oryza sativa* L. cv. KDML105) were surface sterilized by 70% (v/v) ethanol for 1 min followed by 15 min in 5% (w/v) sodium hypochlorite and thoroughly rinsed with sterile distilled water before transferring into a sterile moist chamber and incubated at room temperature in the dark for 7 days. Roots of seedlings were cut into the same length and individually immersed into sterile glass beakers containing 10^8^ spores mL^−1^ of either *S*. *venezuelae*/pIJ86 or *S*. *venezuelae*/pIJ86-*acdS* and incubated for 24 h. Seedlings were then re-located to a moist sponge support for 1 day before transferring to a 20-L container filled with half-Yoshida solution (YS)^54^ for 14 days. Next, salt stress was introduced by replacing the nutrient solution with YS supplemented with 150 mM NaCl and further incubated for 7 days. The pH of nutrient solution was maintained between 5.0–5.5 throughout the growth period. A positive control of non-salt stressed rice was grown under the same conditions without NaCl treatment. Growth parameters of non-salt and salt-stressed rice plants at 7 days were determined for root and shoot lengths, fresh (FW) and dry (DW) weights.

### Analysis of ethylene level and proline accumulation

Ethylene emission was analyzed by the method of Cristescu, *et al*.^55^ 7-day rice plants were placed in a 550 mL bottle tightly sealed with a rubber septum and left for 1 h. Fifty millilitres of headspace air was sampled and analyzed for ethylene by gas chromatography (GC 7890A, Agilent Technologies, USA) packed with a Poropak-N column at 60 °C, equipped with a flame ionization detector. The amount of ethylene emission was calculated as nmol of ethylene g^−1^ FW h^−1^ by comparison to a standard curve generated with pure ethylene.

For proline content, fresh leaf samples (50 mg) were immediately homogenized with liquid nitrogen. The powder was mixed with 3% (v/v) sulfosalicylic acid and centrifuged. The aqueous solution was mixed with ninhydrin solution and glacial acetic acid following the protocol described above.

### Analysis of total chlorophyll and relative water content (RWC)

Total chlorophyll was measured according to the method of Porra, *et al*.^56^. Fresh leaf samples (50 mg) were immediately homogenized with liquid nitrogen. The powder was dissolved in DMSO and centrifuged at 4 °C for 10 min. Absorbance was measured at 645 and 663 nm by spectrophotometry. Total chlorophyll content was calculated based on chlorophyll equations of Arnon^57^.

RWC was determined according to the method of Mostofa and Fujita^58^. Leaf fresh weight was measured and soaked in distilled water for 6 h to determine a turgid weight. The leaves were then dried at 60 °C for 72 h to determine a dry weight. RWC was calculated from each weigh according to Smart and Bingham^59^.

### Determination of Na^+^ and K^+^ contents

Na^+^ and K^+^ contents were analyzed using an atomic absorption spectrophotometer according to the method of Johnson and Ulrich^60^ at The Soil-Fertilizer-Environment Scientific Development Project, Department of Soil Science, Faculty of Agriculture, Kasetsart University. The concentrations of Na^+^ and K^+^ were quantified and calculated as mg g^−1^ DW.

### Analysis of lipid peroxidation and reactive oxygen species (ROS) staining

Lipid peroxidation of leaf samples was estimated by measuring the amount of malondialdehyde (MDA) by a colorimetric method^61^. Fresh leaf samples (50 mg) were immediately homogenized with liquid nitrogen and mixed with 80% ethanol followed by centrifugation. The aqueous solution was mixed with either (i) −TBA solution [20% (w/v) trichloroacetic acid and 0.01% butylated hydroxytoluene], or (ii) +TBA solution (0.65% TBA in −TBA solution). Samples were mixed vigorously and heated at 95 °C for 1 h, cooled on ice and centrifuged. The TBA-MDA complex absorbance was measured at 400, 523 and 600 nm by spectrophotometry. The MDA level was calculated as described by Hodges, *et al*.^61^.

ROS staining of leaf samples was detected using nitrotetrazolium blue chloride (NBT) and 3,3′-diaminobenzidine (DAB) for superoxide and hydrogen peroxide, respectively following the protocol described by Kumar, *et al*.^62^. Leaf samples were separately immersed in 25 mL 2.5 mM NBT staining solution (pH 7.5) and 5 mM DAB staining solution (pH 3.8) for 24 h at room temperature in the dark. The leaves were then decolorized by boiling in 95% (v/v) ethanol for 30 min and further immersed in 60% glycerol for 16 h before color detection.

### Statistical analysis

Data were subjected to statistical analysis using standard ANOVA and Tukey’s multiple range tests of SPSS (version 18.0). Data were presented as mean ± S.E. calculated from four plants per treatment in three different replicates, with a different letter indicating statistical significance at *p* < 0.05. ACC deaminase activity and gene expression ratio data were analysed statistically using a t test at *p* < 0.05. The values represented the mean ± S.E. of three replicates and an asterisk represents a statistically-significant change in expression.

## Supplementary information

Supplementary Information
