# Supplementary material for: Modulation of salt tolerance in Thai jasmine rice (Oryza sativa L. cv. KDML105) by Streptomyces venezuelae ATCC 10712 expressing ACC deaminase
Source: Sci Rep. 2019 Feb 4;9:1275. doi: 10.1038/s41598-018-37987-5 (PMC6361907; doi:10.1038/s41598-018-37987-5)
Supplement: Supplementary file 1 — Supplementary Information [file 41598_2018_37987_MOESM1_ESM.pdf]

Supplementary Information

Modulation of salt tolerance in Thai jasmine rice (*Oryza sativa* L. cv. KDML105) by *Streptomyces venezuelae* ATCC 10712 expressing ACC deaminase

Suranan Yoolong, Worarat Kruasuwan, Huyền Thị Thanh Phạm, Ratchaniwan Jaemsaeng, Chatchawan Jantasuriyarat and Arinthip Thamchaipenet*

Department of Genetics, Faculty of Science, Kasetsart University, Bangkok, Thailand

*Correspondence and requests for materials should be addressed to A.T.
(E-mail: arinthip.t@ku.ac.th)

Supplementary Figures

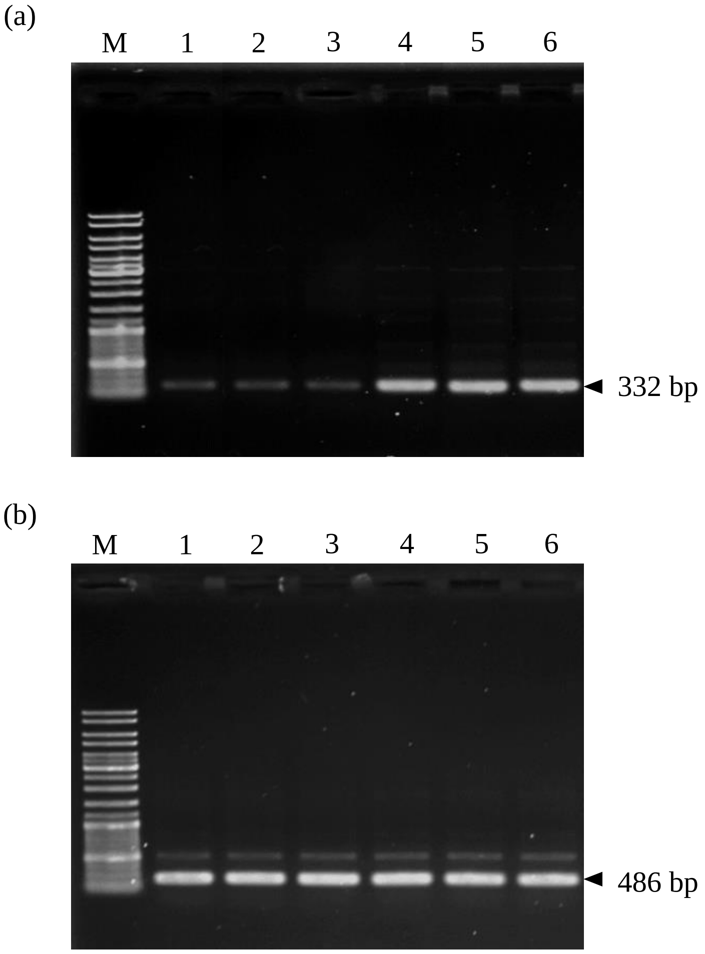

Supplementary Fig. S1 Transcriptional analysis of *acdS* (a) and *hrdB* (b) genes of *S. venezuelae* and its overexpressing mutant by semi-quantitative RT-PCR. Lane M, 1 kb ladder; Lane 1-3, *S. venezuelae*/pIJ86; Lane 4-6, *S. venezuelae*/pIJ86-*acdS*.

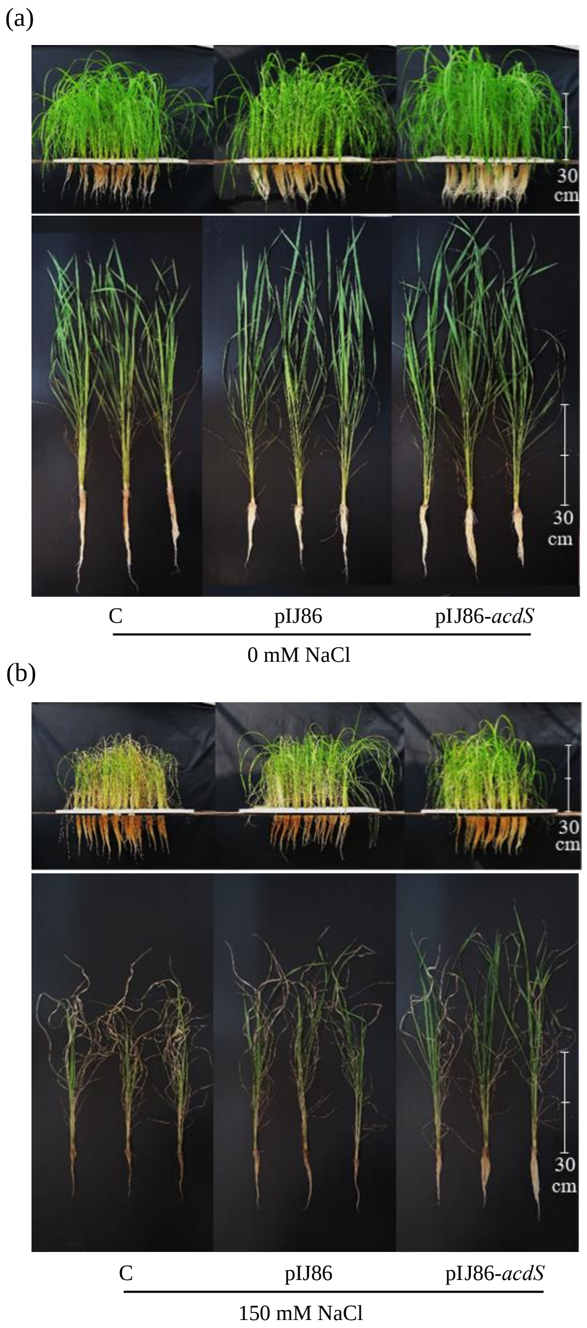

Supplementary Fig. S2 Effect of ACC deaminase-producing *Streptomyces venezuelae* and its overexpressing mutant on the physiology of rice KDML105 after 7 days of non-salt (a) and salt stress (b) treatments under hydroponic conditions. C, uninoculated rice control; pIJ86, rice inoculated with *S. venezulae/*pIJ86; pIJ86-*acdS*, rice inoculated with *S. venezulae*/pIJ86-*acdS*.

Supplementary Tables

Supplementary Table S1 Plant growth promoting (PGP) traits of *Streptomyces venezuelae*

**PGP-traits**	
ACC deaminase	364.21 ± 19.28 nmol α-keto.mg protein^{-1}.h^{-1}
IAA production	21 ± 1.02 μg.mL^{-1}
Proline accumulation	
0% NaCl	21.12 ± 0.30 μM
1% NaCl	25.71 ± 0.39 μM
2% NaCl	30.33 ± 0.59 μM
3% NaCl	36.66 ± 0.24 μM

Supplementary Table S2 ACC deaminase activity of *Streptomyces venezuelae* and its overexpressing mutant grown in MM supplemented with 0.3 mM ACC at 24, 48, 72 and 96 h. The values show the mean ± S.E. of three replicates and an asterisk (*) represents statistically significant different activity (t test, p<0.05). pIJ86, *S. venezuelae*/pIJ86; pIJ86-*acdS*, *S. venezuelae*/pIJ86-*acdS.*

Strain	ACC deaminase activity (nmol α-keto mg protein^{-1} h^{-1})			
	24 h	48 h	72 h	96 h
pIJ86	189.18 ± 10.98	334.70 ± 14.02	364.21 ± 19.28	355.21 ± 12.28
pIJ86-*acdS*	659.68 ± 11.48*	1,465.68 ± 25.34*	1,820.10 ± 13.05*	1,783.10 ± 40.05*

Supplementary Table S3 Semi-quantitative RT-PCR expression profile of *acdS* in *S. venezuelae*/pIJ86 (pIJ86) and *S. venezuelae*/pIJ86 (pIJ86-*acdS*). The expression ratio of *acdS* gene was normalized against the expression of a housekeeping gene, *hrdB*. The values show the mean ± S.E. of three replicates and an asterisk (*) represents statistically-significant different activity (t test, p<0.05).

Strain	Expression ratio
pIJ86	0.351 ± 0.001
pIJ86-*acdS*	0.950 ± 0.006*

Supplementary Table S4 Plant growth parameters of rice KDML105 associated with and without ACC deaminase-producing *Streptomyces venezuelae* and the ACC deaminase-overexpressing mutant under non-salt and salt stress treatments. The values show the mean ± S.E. of twelve replicates and different letters are significantly different (Tukey's test, p<0.05). C, uninoculated rice control; pIJ86, rice inoculated with *S. venezuelae*/pIJ86; pIJ86-*acdS*, rice inoculated with *S. venezuelae*/pIJ86-*acdS*; ND, not detected.

Plant growth parameters	0 mM NaCl			150 mM NaCl		
	C	pIJ86	pIJ86-*acdS*	C	pIJ86	pIJ86-*acdS*
Length (cm)						
Shoot	67.17 ± 1.06^{bc}	78.75 ± 2.54^{a}	80.75 ± 1.78^{a}	59.92 ± 1.29^{d}	66.25 ± 0.39^{c}	72.58 ± 0.50^{b}
Root	19.08 ± 0.66^{ab}	20.75 ± 0.60^{a}	20.50 ± 0.44^{a}	15.08 ± 0.29^{d}	16.92 ± 0.23^{c}	18.00 ± 0.17^{bc}
Fresh weight (g plant^{-1})						
Shoot	1.09 ± 0.04^{b}	1.62 ± 0.06^{a}	1.56 ± 0.04^{a}	0.52 ± 0.03^{d}	0.84 ± 0.02^{c}	1.07 ± 0.02^{b}
Root	0.18 ± 0.00^{b}	0.22 ± 0.01^{a}	0.22 ± 0.01^{a}	0.13 ± 0.01^{c}	0.21 ± 0.01^{a}	0.02 ± 0.01^{a}
Dry weight (g plant^{-1})						
Shoot	0.16 ± 0.01^{b}	0.23 ± 0.01^{a}	0.23 ± 0.01^{a}	0.11 ± 0.01^{c}	0.17 ± 0.005^{b}	0.20 ± 0.00^{a}
Root	0.03 ± 0.00^{c}	0.04 ± 0.00^{a}	0.04 ± 0.00^{a}	0.02 ± 0.00^{d}	0.03 ± 0.00^{bc}	0.04 ± 0.00^{ab}
Relative water content (%)	96.06 ± 1.14^{a}	96.87 ± 1.01^{a}	96.79 ± 1.08^{a}	64.11 ± 2.39^{c}	89.08 ± 1.81^{b}	92.93 ± 0.66^{b}
Ethylene emission (µmoles g^{-1} FW h^{-1})	0.92 ± 0.04^{c}	0.69 ± 0.02^{d}	0.68 ± 0.03^{d}	1.98 ± 0.10^{a}	1.24 ± 0.07^{b}	0.93 ± 0.04^{c}
Proline content (µmole g^{-1} FW)	35.50 ± 1.58^{d}	34.66 ± 1.28^{d}	39.33 ± 3.34^{d}	119.85 ± 4.25^{c}	144.68 ± 7.58^{b}	168.29 ± 9.07^{a}
Total chlorophyll (mg g^{-1} FW)	0.10 ± 0.02^{d}	0.17 ± 0.03^{a}	0.17 ± 0.00^{a}	0.13 ± 0.00^{c}	0.15 ± 0.01^{b}	0.15 ± 0.00^{b}

Supplementary Table S4 (Cont.)

	0 mM NaCl			150 mM NaCl		
	C	pIJ86	pIJ86-*acdS*	C	pIJ86	pIJ86-*acdS*
MDA (nmol g^{-1} FW)	28.79 ± 1.50^{c}	27.48 ± 1.23^{c}	28.21 ± 1.26^{c}	42.63 ± 1.10^{a}	33.45 ± 1.38^{b}	32.88 ± 1.77^{b}
Na^{+}content (mg g^{-1} DW)	0.63 ± 0.09^{c}	0.70 ± 0.03^{c}	0.77 ± 0.03^{c}	35.53 ± 3.94^{a}	27.83 ± 5.76^{ab}	24.83 ± 2.18^{b}
K^{+}content (mg g^{-1} DW)	29.00 ± 6.14^{b}	38.83 ± 3.99^{a}	40.40 ± 4.30^{a}	12.50 ± 2.15^{c}	27.47 ± 1.83^{b}	33.00 ± 2.96^{ab}
CFU g root FW^{-1}	ND	$2.45×10^{4}$	$6.03×10^{3}$	ND	$7.19×10^{4}$	$2.74×10^{4}$
